# A randomized controlled trial of Eye Movement Desensitization and Reprocessing (EMDR) Therapy in the treatment of fibromyalgia

**DOI:** 10.3389/fpsyt.2024.1286118

**Published:** 2024-05-21

**Authors:** Zeynep Zat Çiftçi, Dursun Hakan Delibaş, Taciser Kaya, Duygu Geler Külcü, Aylin Sarı, Hüseyin Nazlıkul, İlkem Coşkun Topsakal, Yunus Emre Aydın, Önder Kavakçı, Canan Savran, Emre Konuk

**Affiliations:** ^1^Institute for Behavioral Studies, Adult and Family Department, Istanbul, Türkiye; ^2^Department of Psychiatry, Izmir Bozyaka Training and Research Hospital, Izmir, Türkiye; ^3^Department of Physical Medicine and Rehabilitation, Izmir Bozyaka Training and Research Hospital, Izmir, Türkiye; ^4^Physical Medicine and Rehabilitation, Yeditepe University Hospital, Istanbul, Türkiye; ^5^Physical Medicine and Rehabilitation, Erenköy Physical Therapy and Rehabilitation Hospital, Istanbul, Türkiye; ^6^Naturel Health, Istanbul, Türkiye; ^7^Department of Psychiatry, Sivas Cumhuriyet University, Sivas, Türkiye

**Keywords:** EMDR, fibromyalgia, RCT, depression, pain

## Abstract

**Background:**

In addition to pharmacological treatment, psychotherapeutic approaches are recommended for the treatment of fibromyalgia. There is a suggestion that eye movement desensitization and reprocessing (EMDR) therapy may be effective. This study aimed to investigate the impact of EMDR therapy on fibromyalgia symptoms, depression, sleep quality, and traumatic stress in fibromyalgia patients through a randomized controlled study (RCT).

**Materials and methods:**

The sample for this study comprised 79 individuals diagnosed with fibromyalgia. Participants were randomly assigned to two groups: the “Treatment as Usual” (TAU) group and the TAU + EMDR group. Prior to the study and at six different time points (before starting the study, at the end of the 5th, 10th, and 15th sessions, 1 month later, and 3 months later), participants completed assessments, including the Fibromyalgia Impact Questionnaire (FIQ), Visual Analog Scale (VAS), Fibromyalgia ACR 2010 Diagnostic Criteria [Widespread Pain Index (WPI) and Symptom Severity Scale (SSS)], Beck Depression Inventory (BDI), Pittsburgh Sleep Quality Index (PSQI), and Trauma Symptom Checklist-40 (TSC-40).

**Results:**

There were no differences in the sociodemographic variables between the study and experimental groups. Analysis of variance revealed a statistically significant group effect on VAS (p = 0.019), WPI (p = 0.018), BDI (p = 0.019), and TSC-40 (p = 0.21). After applying Bonferroni correction, EMDR was found to be effective for VAS, WPI, SSS, BDI, PSQI, and TSC-40 (p <0.05).

**Conclusion:**

The results of the current study suggest that EMDR therapy is a viable alternative treatment for fibromyalgia. We believe these findings offer robust evidence supporting the efficacy of EMDR therapy in treating fibromyalgia, particularly in the context of a randomized controlled trial (RCT). The application of EMDR therapy for the treatment of patients with fibromyalgia is likely to be beneficial.

**Clinical trial registration:**

ClinicalTrials.gov, identifier NCT06265194.

## 1 Introduction

Fibromyalgia, the most commonly encountered chronic widespread pain (CWP) condition, is characterized by clinically unexplained chronic pain, allodynia (pain produced by innocuous stimuli), and fatigue persisting for at least 3 months (1, 2). Other essential symptoms of fibromyalgia include chronic pain, sleep problems, fatigue, and emotional issues (3). Its prevalence ranges from 2% to 4% (4). While the organic and medical etiology of fibromyalgia is not well documented, it is acknowledged that it negatively impacts the quality of life. The effectiveness of established treatments for fibromyalgia is limited, prompting the recommendation of non-pharmacological interventions for its management (3). This suggestion is based on the understanding that childhood traumas, stressful life events, and stress induced by pain play a significant role in the development and exacerbation of fibromyalgia pain (5–7).The literature indicates that in addition to personalized physical exercise, treatments such as cognitive–behavioral therapy, mindfulness-based stress reduction, meditation, hydrotherapy, or a combination of these methods offer hope for patients diagnosed with fibromyalgia (8).

Eye movement desensitization and reprocessing therapy (EMDR) is a psychotherapeutic approach that has been extensively studied through scientific research, demonstrating its effectiveness in treating post-traumatic stress disorder, depression, anxiety, and various disorders related to adverse life events (9, 10). The foundation of eye movement desensitization and reprocessing therapy (EMDR) is rooted in the Adaptive Information Processing (AIP) model. According to this model, the mind naturally processes and integrates information into a healthy manner. However, traumatic experiences can disrupt this process, leading to the maladaptive storage of traumatic memories. EMDR, guided by the AIP model, aims to facilitate the reprocessing and integration of traumatic memories through bilateral stimulation. The overarching goal of EMDR is to achieve emotional healing and symptom reduction by assisting individuals in processing traumatic memories in a healthier manner (11). During traumatic experiences, painful stimuli are not only stored as physical sensations, but also as images, cognitions, and emotions. The nociceptive senses and emotional reactions are interconnected, and traumatic memories often involve affective elements that significantly contribute to chronic pain and stress (12). The adaptive reprocessing of these maladaptive stored memories can lead to symptom recovery by promoting the integration of distressing memories. According to the AIP model, perception associated with traumatic memories, along with their somatic and affective components, undergoes reprocessing through bilateral stimulation. This process facilitates cortical integration of memories, ultimately contributing to their resolution. Changes in the emotional dimensions of pain can influence the recall and reproduction of pain within the nervous system by altering the pain pathways. After successful treatment with EMDR, emotional distress is relieved, negative beliefs are reformulated, and physiological arousal is reduced (13). Although EMDR therapy is widely recognized for its efficacy in treating PTSD, numerous studies have been highlighted its effectiveness in addressing various issues (9). Researchers have extended the application of EMDR to the treatment of fibromyalgia patients, aligning with the recommendation of psychotherapeutic methods and evidence indicating the potential role of traumatic experiences in the development and maintenance of chronic pain (14, 15). According to this perspective, the Adaptive Information Processing (AIP) model conceptualizes chronic pain as a form of recurring “trauma,” with the traumatic event comprising recurring emotions and constant physical symptoms. It has been hypothesized that chronic pain may be reduced or eliminated through the processing of traumatic experiences using EMDR Therapy. Despite the promising outcomes of these studies, randomized controlled trials are necessary to further establish the efficacy of EMDR in the treatment of fibromyalgia.

Based on this information and the hypothesis that EMDR may be useful in relieving pain and complaints in patients with fibromyalgia, this study aimed to evaluate the effectiveness of a specific EMDR Fibromyalgia Protocol. Additionally, this study aimed to reveal the effects of EMDR therapy on depression, sleep quality, and traumatic stress symptoms in individuals with fibromyalgia.

## 2 Material and methods

### 2.1 Participant

The sample for this multicenter Randomized Controlled Trial (RCT), conducted between October 2015 and October 2018, consisted of patients diagnosed with fibromyalgia. Inclusion criteria were a) diagnosis of fibromyalgia, b) age between 18 and 65, c) compliance with routine medical fibromyalgia treatment, d) cognitive and technical competence to meet the working conditions, and e) volunteering to participate in the study. Exclusion criteria included a) receiving any psychotherapy, b) presence of psychiatric disorders such as schizophrenia or bipolar affective disorder, c) no other physical or psychological targets apart from trauma that need to be addressed before fibromyalgia pain (e.g., suicide, domestic violence, etc.), and d) presence of an organic cause that can cause pain.

**Figure 1:**
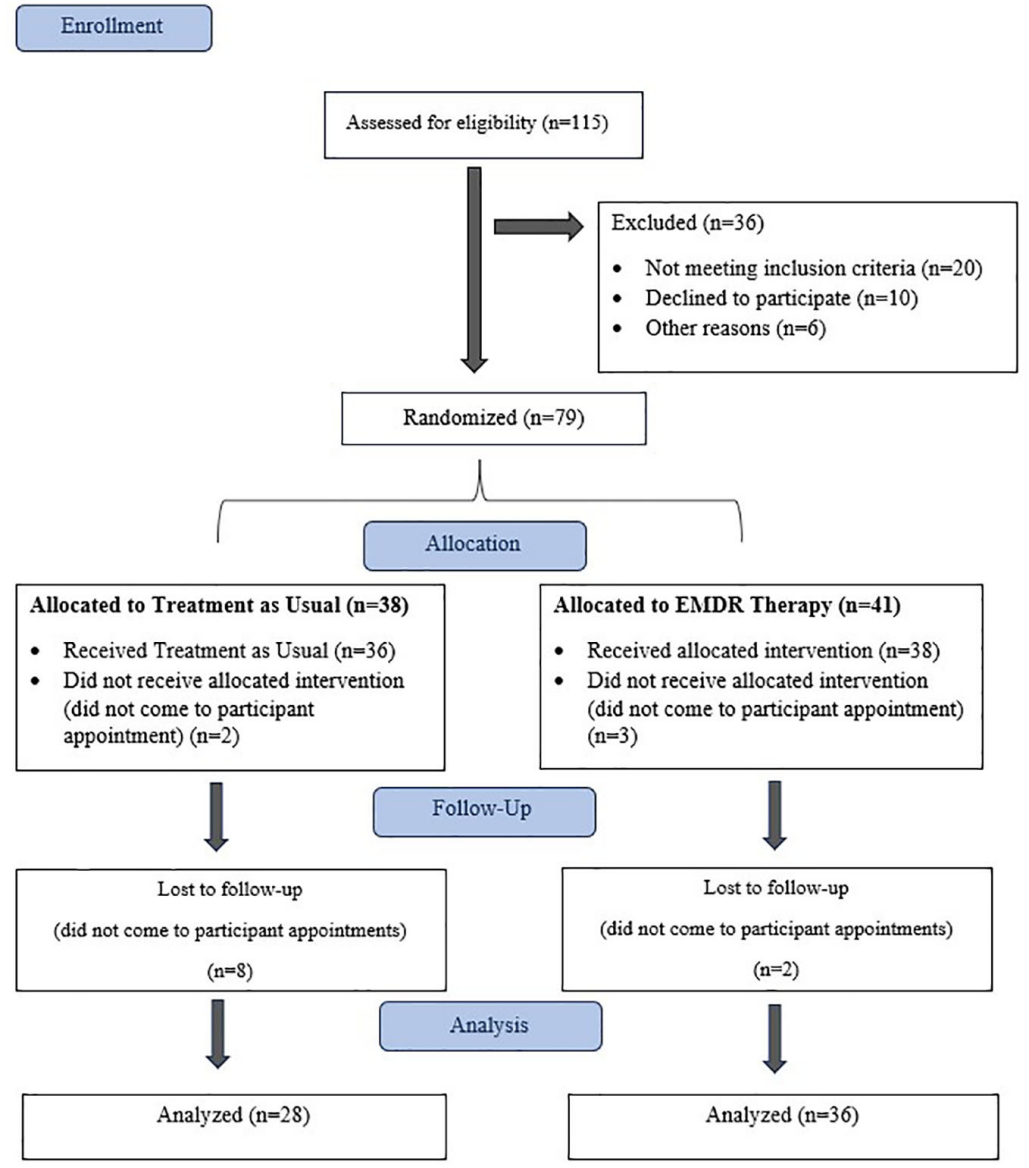
Flowchart of the study.

A total of 115 patients were initially referred for the study by rheumatologists. Following the initial evaluation, 36 patients were excluded, leaving 79 patients who were randomized. However, during the study process, 38 participants from the “Treatment As Usual” (TAU) group and 41 participants from the EMDR therapy group were excluded for various reasons. Consequently, data from 28 and 36 participants in the TAU and EMDR therapy groups, respectively, were included in the final analysis (**Figure 1**).

### 2.2 Study design

Participants who sought treatment at the rheumatology outpatient clinic were diagnosed with fibromyalgia by experienced clinicians following necessary examinations and tests (16). Routine fibromyalgia treatment, referred to as Treatment as Usual (TAU) in this study, was initiated in the outpatient clinic. TAU is a treatment designed by rheumatologists in accordance with the current rheumatology guidelines (16). Subsequently, patients meeting the inclusion criteria were referred to the study, informed of their details, and evaluated based on predefined inclusion and exclusion criteria. Eligible participants were randomized into two groups: one receiving TAU treatment (control group) and the other receiving both TAU and EMDR treatment (experimental group). Participants underwent assessments using various scales, including the Fibromyalgia Impact Questionnaire (FIQ), Visual Analog Scale (VAS), Fibromyalgia ACR 2010 Diagnostic Criteria (Widespread Pain Index [WPI] and Symptom Severity Scale [SSS]), Beck Depression Inventory (BDI), Pittsburgh Sleep Quality Index (PUQI), and Trauma Symptom Checklist-40 (TSC-40). Assessments were conducted before study initiation, at the end of the 5th, 10th, and 15th sessions, and at 1st month and 3rd months post-treatment, totaling six different times. Scale scores were statistically according to the respective instructions.

To conduct this study, therapists experienced in EMDR Therapy were informed about the project and invited to participate. The selection criteria for therapists included the completion of EMDR Level I Training. The therapists underwent training using the EMDR Fibromyalgia Protocol (17). Following their preparation, therapists commenced working with participants who were randomly allocated to either the control or experimental groups. The maximum number of EMDR sessions was 15. Regular supervision meetings were provided to all therapists throughout this period.

Therapists delivering EMDR Therapy provided psychotherapeutic services to clients in the experimental group using EMDR Fibromyalgia Treatment. All psychological measurements were administered to clients by a research assistant. Upon completion of the scales, they were returned to the research assistant. Data were received and entered into the system on a weekly basis by a research assistant to ensure the security of the data collection process.

#### 2.2.1 EMDR fibromyalgia protocol

The fibromyalgia protocol applied to the patients in this study was developed specifically for fibromyalgia by Konuk et al. In addition to the article, the details of the protocol and the study sheet are presented supplementary. After a short introduction, the participants were informed about the protocol, their consent was obtained for registration, and a visual, tactile, and audio bilateral stimulus that would be used in the application was introduced. EMDR was then applied, as described below:

Phase 1: History Taking

- Medical Assessment: Fibromyalgia was diagnosed by a physician. Never accept a client without being diagnosed by a physician.
- Psychiatric Assessment: Psychiatric consultations should be conducted. If any medication is required, it needs to be administered as usual. Some clients have “psychiatric” problems in addition to chronic musculoskeletal pain. The way the clinician chooses to begin and work with them depends on clinical judgment. The protocol suggests that the clinician begins with and sticks to negative life events (traumas) that she/he thinks are related to the chronic musculoskeletal pain the client has. In approximately 8–15 sessions, the clinician will see where the treatment is and then decide together with the client if they will work with other traumas and life experiences.
- Psychological Assessment: It is advised to use scales that are used in this article to assess pain and its effects on the client’s functioning.
- Intake as Usual: They can get anything you need to know about the client.
- Case Formulation: Clients come to therapy to talk about their complaints and hope to solve them. They did not speak the language of trauma, EMDR, or AIP. They simply complained and asked for solutions. In this case, they want to “get rid of” the pain.

It is the job of the EMDR therapist to formulate problems and symptoms in terms of trauma, EMDR, and AIP. You will have to use AIP (theory) to connect trauma with chronic musculoskeletal pain.

The premise is that chronic musculoskeletal pain begins with trauma or a series of traumas and is maintained by events interpreted (memory networks) as trauma (negative life events).

At this point, the guide is the standard protocol: past, present (triggers), and future (future template). After gathering the presenting problems and traumatic memories, the clinician performs a case formulation.

Phase 2: Preparation

At this stage, therapists prepare the client for EMDR. The rules and procedures of the Standard Protocol are applied. Resourcing and stabilization are performed. Light-stream and spiral techniques can also be used.

Inform the client about the treatment process and EMDR.

Phase 3: Assessment

Clinician makes treatment plan and clinical decisions on how to choose the target memory. At this point, the clinician has most of the information you need to plan your treatment. The clinician may feel free to obtain any other necessary information. The general strategy is to first focus on targets (memories) directly related to fibromyalgia pain.

Phases 4–8: Desensitization to Re-evaluation

Here the rules and procedures of the Standard Protocol are applied.

The client works on pain-related memories and triggers, and then on a Future Template. This takes up to 15 sessions. As general rules, sessions are 75–90 min.

### 2.3 Measurements

#### 2.3.1 Demographic information

At the initial stage, patient characteristics, including sex, age, marital status, job status, and education level, were determined for patients diagnosed with fibromyalgia in both the experimental and control groups. A questionnaire comprising variables associated with fibromyalgia according to scientific sources was prepared and administered to both groups.

#### 2.3.2 Fibromyalgia Impact Questionnaire

The FIQ was first developed in the 1980s by Drs. Carol Burckhardt, Sharon Clark, and Robert Bennett (18). During the scale’s development phase, the clinical characteristics of fibromyalgia were recently described, and the FIQ was an initial effort to assess problems related to fibromyalgia and therapy responses. The FIQ was first used in 1991 and has since been widely acknowledged as a measure of therapeutic efficacy. The physical impairment items on the scale are rated on a 4-point Likert scale ranging from “always” to “never.” A Turkish validity and reliability study of the scale was conducted by Sarmer et al. in 2000 (19), reporting an internal consistency of 0.72.

#### 2.3.3 Visual Analog Scale

VAS is employed to convert non-numerically measurable values into numerical representations. Specifically designed to measure the intensity of pain in adult populations with rheumatic diseases, this scale comprises 45 pain zones. Participants rated their pain intensity on a scale of one to five stars, excluding areas without pain (marked as 0 points). The maximum score on this scale is calculated as 45 ∗ 5 = 225 points. Once the total points are obtained, they are converted into a system where 100 represents the maximum score. The advantages of the test include its language-free nature and ease of application.

#### 2.3.4 Fibromyalgia ACR 2010 Diagnostic Criteria

The Fibromyalgia ACR 2010 Diagnostic Criteria are a diagnostic measure for fibromyalgia, consisting of two parts assessing the extent of widespread pain and symptom severity. The Widespread Pain Index (WPI) evaluates the number of areas with pain complaints lasting more than a week, encompassing 19 bodily areas such as the shoulder girdle, upper arm, forearm, hip, thigh, leg, chin, chest, abdomen, back, waist, and neck. Each aching area was assigned one point, resulting in a total score ranging from 0 to 19.The Symptom Severity Scale (SSS) examines symptoms in four sections: fatigue, waking up tired, cognitive problems, and somatic symptoms. The overall score on this scale ranges from 0 to 12 points. The reliability analysis of the Turkish version, conducted by Yanmaz, Atar, and Bicer (20), has demonstrated the measure’s reliability and validity. Cronbach’s alpha for the scale ranged from.76 to.77 for the total score.

#### 2.3.5 Beck Depression Inventory

The Beck Depression Inventory was developed by Aaron T. Beck in 1961 (21) is a widely used 21-question multiple-choice self-report scale designed to measure the severity of depression. Each question is rated on a scale from 0 to 3, with higher total scores indicating greater symptom severity. Reliability and validity studies have been conducted for the Turkish version by Hisli (22), and the reported values were high.

#### 2.3.6 Pittsburgh Sleep Quality Index

The Pittsburgh Sleep Quality Index is a self-report scale that assesses sleep quality and impairment over a one-month period. Validity and reliability studies of the Turkish population were conducted by Agargun et al. (23). The questionnaire comprised 19 questions, evaluating seven sub-dimensions: subjective sleep quality, sleep latency, sleep duration, habitual sleep efficiency, sleep disturbance, use of sleeping pills, and daytime dysfunction. Each response was scored between 0 and 3, based on the symptom frequency. The total PSQI score, obtained by summing the scores of the seven components, ranged from 0 to 21. Higher values indicate poor sleep quality and a higher level of sleep disturbance (24).

#### 2.3.7 Trauma Symptom Checklist-40

The Trauma Symptom Checklist-40 assesses trauma symptoms in both childhood and adulthood using six categories. This self-report scale includes subscales of dissociation, anxiety, depression, sexual abuse, trauma index, sleep disturbance, and sexual problems. Developed by Briere and Runtz in 1989 (25), the TSC-40 is comprised of 40 questions and is designed to evaluate symptoms over the past month.

### 2.4 Data analysis

In this part of the study, the frequencies and percentages of discrete variables were established for both groups. Then, a chi-square test of independence was performed to ensure goodness-of-fit between the experimental and control groups. Chi-square analysis was used to check whether the group factors and sociodemographic variables were related. The chi-square tests of independence yielded no significant results; thus, the experimental and control groups were balanced regarding demographic variables.

After the descriptive statistics were calculated for the continuous dependent variables of both groups, the normality of distribution was examined using the Kolmogorov–Smirnov test. Statistical significance was set at p >0.05. When the normality of distribution was established, we used parametric statistical techniques to determine the differences between the experimental and control groups in terms of continuous variables in our study.

An independent groups t-test was performed to compare the experimental and control groups for the continuous independent variables of the study.

After the equation of the experimental and control groups in terms of pre-tests, a 2 × 6 two-factor repeated measures ANOVA was conducted to test the hypotheses built for each of the continuous variables. First, descriptive statistics were calculated for each of the continuous variables from the group ∗ time perspective. Later, Levene’s test was conducted to examine the homogeneity of the variances of the pre-test, end of the 5th, 10th, and 15th sessions, and 1st and 3rd-month follow-up test scores. Subsequently, Box’s M test was performed to establish that the covariance matrices of the experimental and control groups were equal. Then, the significance of the F values of the group (experimental/control), measurement time pre-test, end of the 5th, 10th, and 15th sessions, and the 1st and 3rd-month follow-up tests), and group ∗ measurement time were examined. Since ANOVA results for the group and measurement time were statistically significant, to determine the source of the variance between groups and to examine the basic effect of time for each group, one-way repeated ANOVA was applied to the data (separately for the experimental and the control groups), where a difference was observed between groups, Bonferroni corrections were made to determine the differences between the two groups. Due to experimental design of this study, all results were analyzed with a one-way analysis of variance and the statistical significance was established as p >0.05. All statistical analyses were performed using SPSS for Windows.

## 3 Results

### 3.1 Demographic information

A comparison of the participants’ sociodemographic information is presented in **Table 1**. No statistical difference was found between the experimental and control groups (p >0.05). To test the hypotheses regarding this study, first, the arithmetic means and the standard deviations were calculated for the FIQ, VAS), WPI, SSS, BDI, PUQI, and TSC-40 scores in the pre-test, end of the 5th, 10th, and 15th sessions, and the 1st and 3rd-month follow-up measurements (**Table 2**).

**Table 1:** Demographic characteristics of groups at baseline.

Characteristic	Total (n = 64)	Experimental (n = 36)	Control (n = 28)	Analysis		
				Chi-square	df	p
**Gender**				.065	1	.795
** Female**	60 (93.8%)	34 (94.4%)	26 (92.9%)			
** Men**	4 (6.3%)	2 (5.6%)	2 (7.1%)			
**Education**				10.342	3	.051
** Primary School**	20 (31.3%)	4 (11.1%)	16 (57.1)			
** Middle School**	6 (9.4%)	2 (5.6%)	4 (14.3%)			
** High School**	19 (29.7%)	14 (38.9%)	5 (17.9%)			
** University**	19 (29.7%)	16 (44.4)	3 (10.7%)			
**Marital status**				6.89	2	.075
** Married**	47 (73.4)	23 (63.9%)	24 (85.7%)			
** Single**	13 (20.3%)	10 (27.8%)	3 (10.7%)			
** Divorce**	4 (6.3%)	3 (8.3%)	1 (3.6%)			
**Working status**				6,89	2	.075
** working**	18 (28.1%)	14 (38.9%)	4 (14.3%)			
** Not-working**	34 (53.1%)	13 (36.1%)	21 (75.0%)			
** retired**	12 (18.8%)	9 (25.0%)	3 (10.7%)			
**Fibromyalgia in the family**				3.426	2	.180
** Yes**	14 (21.9%)	8 (22.2%)	6 (21.4%)			
** No**	46 (71.9%)	24 (66.7%)	22 (78.6%)			
** I don’t know**	4 (6.3%)	4 (11.1%)	0 (.0%)			
**Mental health disorder family**				.311	1	.577
** Yes**	23 (35.9%)	14 (38.9%)	9 (32.1%)			
** No**	41 (64.1%)	22 (61.1%)	19 (67.9%)			
**Mental therapy in family**				1.693	1	.193
** Yes**	24 (37.5%)	16 (44.4%)	8 (28.6%)			
** No**	40 (62.5%)	20 (55.6%)	20 (71.4%)			
**Getting help fibromyalgia**				.805	1	.370
** Yes**	35 (54.7%)	15 (41.7%)	20 (71.4%)			
** No**	29 (45.3%)	21 (58.3%)	8 (28.6%)			
**Mean age**				-1.120	62	.267
	43.99 (12.46)	42.22 (12.70)	45.75 (12.24)			

**Table 2:** Participants’ pre and post and follow-up scale scores.

Scale	Group	Measurement											
		Pre-test		5th Session		10th Session		15th Session		1st month follow-up		3^st^ month follow-up	
		$\bar{\text{X}}$	SD	$\bar{\text{X}}$	SD	$\bar{\text{X}}$	SD	$\bar{\text{X}}$	SD	$\bar{\text{X}}$	SD	$\bar{\text{X}}$	SD
**Visual Analog Scale**	Experimental	31.04	9.82	22.04	8.08	15.29	8.65	12.53	16.78	13.27	18.27	12.18	17.30
	Control	33.26	22.62	25.82	17.36	25.02	16.87	25.61	17.75	28.07	17.58	26.18	17.65
**Fibromyalgia Impact Questionnaire**	Experimental	61.72	13.27	53.29	13.34	40.36	18.88	37.89	18.63	33.71	20.96	36.29	19.03
	Control	55.24	19.80	52.37	13.34	49.19	22.38	48.77	21.47	53.63	19.21	50.51	17.56
**Widespread Pain Index Subscale of Fibro Diagnostic Criteria**	Experimental	12.72	4.55	9.66	5.54	6.94	5.23	6.47	4.63	5.86	4.52	5.36	4.16
	Control	11.00	4.43	10.14	4.18	9.78	5.15	9.35	5.42	9.89	4.32	11.25	4.65
**Symptom Severity Subscale of Fibro Diagnostic Criteria**	Experimental	2.26	0.65	1.91	0.68	1.53	0.77	1.31	0.62	1.35	0.79	1.37	0.63
	Control	1.67	0.79	1.74	0.78	1.67	0.78	1.41	0.94	1.84	0.79	1.77	0.69
**Beck Depression Inventory**	Experimental	42.83	9.82	36.47	8.08	32.78	8.65	29.67	9.64	29.81	9.05	29.53	8.06
	Control	39.50	9.10	39.04	8.86	38.07	9.23	37.18	9.64	38.11	9.09	36.25	8.73
**Trauma Symptom Checklıst-40**	Experimental	31.04	17.86	35.30	14.38	31.36	16.39	26.72	15.11	25.44	15.34	25.80	14.03
	Control	33.26	22.62	39.50	15.64	37.82	17.09	39.52	16.58	36.53	16.55	36.36	17.42
**Pittsburg Sleep Quality Index**	Experimental	11.08	3.75	9.66	8.08	7.93	3.99	7.09	3.99	7.13	3.23	6.98	3.33
	Control	9.50	3.75	9.68	4.42	9.28	4.48	9.85	4.58	9.07	4.10	10.00	4.53

### 3.2 Visual Analog Scale

Two-Factor Repeated Measures ANOVA (2 × 6): The variance analysis was conducted on the mean scores of the pre-test, end of the 5th, 10th, and 15th sessions, and follow-up measurements revealed a significant group effect for VAS scores in both the experimental and control groups (F_(1, 62)_ = 5.768; p <0.05) (**Table 3**). Additionally, a significant difference was observed between measurements taken at different time points (pre-test, end of the 5th, 10th, and 15th sessions, 1st and 3rd-month follow-up) (F_(5,310)_= 17.471; p <0.001). The interaction effect (group ∗ measurement) also reached significance (F_(5, 310)_ = 5.061; p <0.001).

**Table 3:** 2 × 6 Two-factor repeated measures ANOVA for pre-post (1–2–3) and follow up (1–2) tests scale scores of experimental and control groups.

Scale	Resource of Variance	Sum Squares	df	Mean Square	F	P
**Visual Analog Scale**	**Group(E/C)**	8,711.099	1	8711.099	5.768	**0.019**
	**Error**	93,633.742	62	1510.222		
	**Measure (pre/post/f)**	7,994.433	5	1598.887	17.471	**<0.001**
	**Group ∗ Measure**	2,315.705	5	463.141	5.061	**<0.001**
	**Error**	28,369.361	310	91.514		
**Fıbromyalgıa Impact Questıonnaıre**	**Group(E/C)**	5,667.954	1	5667.954	3.879	0.053
	**Error**	90,592.153	62	1461.164		
	**Measure (pre/post/f)**	12,906.468	5	2581.294	19.119	**<0.001**
	**Group ∗ Measure**	7,535.764	5	1507.153	11.163	**<0.001**
	**Error**	41,853.927	310	135.013		
**Widespread Pain Index Subscale of Fibro Diagnostic Criteria**	**Group(E/C)**	544.380	1	544.380	5.945	**0.018**
	**Error**	5,677.305	62	91.569		
	**Measure (pre/post/f)**	776.181	5	155.236	19.052	**<0.001**
	**Group ∗ Measure**	566.347	5	113.269	13.901	**<0.001**
	**Error**	2,525.890	310	8.148		
**Symptom Severity Subscale of Fibro Diagnostic Criteria**	**Group(E/C)**	0.400	1	0.400	0.188	0.666
	**Error**	131.738	62	2.125		
	**Measure (pre/post/f)**	14.300	5	2.860	12.074	**<0.001**
	**Group ∗ Measure**	12.312	5	2.462	10.395	**<0.001**
	**Error**	73.432	310	0.237		
**Beck Depression Inventory**	**Group(E/C)**	1,922.072	1	1922.072	5.822	**0.019**
	**Error**	20,468.696	62	330.140		
	**Measure (pre/post/f)**	3,167.625	5	633.525	24.163	**<0.001**
	**Group ∗ Measure**	1,483.687	5	296.737	11.318	**<0.001**
	**Error**	8,127.966	310	26.219		
**Trauma Symptom Checklıst-40**	**Group(E/C)**	5,836.439	1	5836.439	5.655	**0.021**
	**Error**	63,994.766	62	1032.174		
	**Measure (pre/post/f)**	1,894.965	5	378.993	3.093	**0.010**
	**Group ∗ Measure**	1,381.046	5	276.209	2.254	**0.049**
	**Error**	37,983.755	310	122.528		
**Pittsburgh Sleep Quality Index**	**Group(E/C)**	147.839	1	147.839	2.412	0.125
	**Error**	3,799.531	62	61.283		
	**Measure (pre/post/f)**	226.503	5	45.301	8.025	**<0.001**
	**Group ∗ Measure**	242.634	5	48.527	8.596	**<0.001**
	**Error**	1,749.954	310	5.645		

In **Table 3**, statistically significant values are shown in bold.

Bonferroni Test: The VAS (end of the 5th session), mean score of the experimental group was found to be significantly higher (p <0.05) than the end of the 10th and 15th sessions, and the 1st and 3rd month follow-up mean scores. However, no significant difference was found between the end of the 5th, 10th, and 15th session VAS scores of the experimental group and the 1st and 3rd month follow-up test scores (p >0.05).

### 3.3 Fibromyalgia Impact Questionnaire

Two-Factor Repeated Measures ANOVA (2 × 6): The analysis indicated that the group effect was not significant for the total FIQ scores in both the experimental and control groups (F_(1, 62)_ = 3.879; p >0.05) (**Table 3**). Additionally, a significant difference was observed between measurements taken at different time points (pre-test, end of the 5th, 10th, and 15th sessions, and 1st and 3rd-month follow-up) (F_(5,310)_ = 19.119; p <0.001). The interaction effect (group ∗ measurement) also reached statistical significance (F_(5, 310)_ = 11.163; p <0.001).

Bonferroni Test: Total FIQ scores at the pretest were found to be significantly higher than scores at the end of the 5th, 10th, and 15th sessions, and at the 1st and 3rd-month follow-up. Similarly, scores at the end of the 5th session were significantly higher than those at the end of the 10th and 15th sessions, and the 1st and 3rd-month follow-up. The means of the end of the 15th session and at the 1st and 3rd-month follow-up test scores were found to be equal. This result suggests that the decrease in scores continued during the follow-up tests. No significant difference was found between the 1st and 3rd-month follow-up test scores. In the control group, one-way variance analysis applied for repeated measures did not show a statistically significant difference (F_(5, 135)_ = 1.970; p >0.05).

### 3.4 Fibromyalgia ACR 2010 Diagnostic Criteria

#### 3.4.1 Widespread Pain Index Subscale

Two-Factor Repeated Measures ANOVA (2 × 3): The analysis revealed a significant group effect for the WPI Subscale scores in both the experimental and control groups (F_(1, 62)_ = 5.945; p <0.05; **Table 3**). However, a significant difference was also observed between the measurements at different time points (pre-test, end of the 5th, 10th, and 15th sessions, and at the 1st and 3rd-month follow-up) (F_(5, 310)_ = 19.052; p <0.001). In this study, the interaction effect (group ∗ measurement) was also significant (F_(5, 310)_ = 13.901; p <0.001).

Bonferroni Test: Statistically significant differences were found in the one-way analysis of variance for repeated measures applied to the experimental group (F_(5, 175)_ = 31.325; p <0.001). Total WPI scores at pre-test were significantly higher than scores at the end of the 5th, 10th, and 15th sessions, and at the 1st and 3rd-month follow-up. Similarly, scores at the end of the 5th session were significantly higher than those at the end of the 10th and 15th sessions, and at the 1st and 3rd-month follow-up. The means of the end of the 15th session and the 1st and 3rd-month follow-up test scores were found to be equal. This result suggests that the decrease in scores continues in the follow-up tests as well. No statistically significant differences were found in the one-way analysis of variance for repeated measures applied to the control group (F_(5, 135)_ = 2.228; p >0.05).

#### 3.4.2 Symptom Severity Subscale

Two-Factor Repeated Measures ANOVA (2 × 3): The analysis revealed a significant group effect for WPI Subscale scores in both the experimental and control groups (F_(1, 62)_ = 5.945; p <0.05; **Table 3**). However, a significant difference was also observed between measurements at different time points (pre-test; end of the 5th, 10th, and 15th sessions; and at the 1st and 3rd-month follow-up) (F_(5, 310)_ = 19.052; p <0.001). In this study, the interaction effect (group ∗ measurement) was also significant (F_(5, 310)_ = 13.901; p <0.001).

Bonferroni Test: Statistically significant differences were found in the one-way analysis of variance for repeated measures applied to the experimental group (F_(5, 175)_ = 31.325; p <0.001). Total WPI scores at pretest were significantly higher than scores at the end of the 5th, 10th, and 15th sessions, and at the 1st and 3rd-month follow-up. Similarly, scores at the end of the 5th session were significantly higher than those at the end of the 10th and 15th sessions, and at the 1st and 3rd-month follow-up. The means of the end of the 15th session, and the 1st and 3rd-month follow-up test scores were found to be equal. This result suggests that the decrease in scores continues in the follow-up tests as well. No statistically significant differences were found in the one-way analysis of variance for repeated measures applied to the control group (F_(5, 135)_ = 2.228; p >0.05).

### 3.5 Beck Depression Inventory

Two-Factor Repeated Measures ANOVA (2 × 6): The analysis of the mean scores of the pre-test, end of the 5th, 10th, and 15th sessions, and the 1st and 3rd-month follow-up measurements for BDI scores in the experimental and control groups demonstrated a significant group effect (F_(1,62)_ = 5.822; p <0.05) (**Table 3**). The experimental and control groups showed a significant difference in their overall BDI Total scores, without discrimination between the pre-test, end of the 5th, 10th, and 15th sessions, and the 1st and 3rd-month follow-up measures. Furthermore, a significant difference was observed between measurements at different time points (pre-test; end of the 5th, 10th, and 15th sessions; and the 1st and 3rd-month follow-up) (F_(5, 310)_ = 24.163; p <0.001). The interaction effect (group ∗ measurement) was also significant (F_(5, 310)_ = 11.318; p <0.001).

Bonferroni Test: The BDI pre-test mean score of the experimental group was significantly higher (p <0.001) than the end of the 5th, 10th, and 15th sessions and the 1st and 3rd-month follow-up mean scores. Regarding the positive effect of the therapy sessions on depression scores, the BDI mean score at the end of the 5th session for the experimental group was significantly higher (p <0.01) than the mean scores at the end of the 5th, 10th, and 15th sessions. However, no significant difference was found between the end of the 15th session BDI scores of the experimental group and the 1st and 3rd-month follow-up test scores (p >0.05).

### 3.6 Trauma Symptom Checklist-40

Two-Factor Repeated Measures ANOVA (2 × 6): The analysis indicated that the group effect was significant for trauma symptom screening scores in both the experimental and control groups (F_(1,62)_ = 5.655; p <0.05) (**Table 3**). There was a significant difference between the average trauma symptom screening scores of the experimental and control groups without any distinction between the pre-test, end of the 5th, 10th, and 15th sessions, and the 1st and 3rd-month follow-up. Moreover, a significant difference was observed between measurements at different time points (pre-test; end of the 5th, 10th, and 15th sessions; and the 1st and 3rd-month follow-up) (F_(5, 310)_ = 3.063; p <0.01). The interaction effect (group ∗ measurement) was also significant (F_(5, 310)_ = 2.254; p <0.05).

Bonferroni Test: TSC-40 (end of the 5th session) mean score of the experimental group was significantly higher (p <0.05) than the end of the 15th session and the 1st and 3rd-month follow-up mean scores. The mean TSC-40 (end of the 10th session) was found to be significantly (p <0.05) higher than that of the 15th session and the 1st-month follow-up. At the end of the 15th session, the mean scores did not differ from the 1st and 3rd-month follow-up scores. In this case, the scores on the follow-up tests remained at the same level as those in the last measurement. No statistically significant differences were found in the one-way analysis of variance for repeated measures applied to the control group (F_(5, 135)_ = 1.090; p >0.05).

### 3.7 Pittsburgh Sleep Quality Index

Two-Factor Repeated Measures ANOVA (2 × 6): The analysis indicated that the group effect was not significant for the total PSQI scores in both the experimental and control groups (F_(1, 62)_ = 2.412; p >0.05) (**Table 3**). However, a significant difference was observed between measurements at different time points (pre-test; end of the 5th, 10th, and 15th sessions; and the 1st and 3rd-month follow-up) (F_(5, 310)_ = 8.025; p <0.001). In this study, the interaction effect (group ∗ measurement) was also significant (F_(5, 310)_ = 8.596; p <0.001).

Bonferroni Test: A statistically significant difference (p <0.001) was found for the experimental group (F_(5, 175)_ = 27.211; p <0.001) in the one-way variance analyses for repeated measures applied to the experimental group. However, no statistically significant differences were found in the one-way analysis of variance for repeated measures applied to the control group (F_(5, 135)_ = 0.424; p >0.05).

## 4 Discussion

This multicenter RCT indicated that the EMDR Fibromyalgia Protocol could be a secure, dependable, and effective approach for treating fibromyalgia. The alleviation of fibromyalgia pain appears to persist even after the conclusion of the therapy sessions, as demonstrated in the follow-up measurements at 1 and 3 months. In summary, patients with fibromyalgia who underwent the EMDR Fibromyalgia Protocol exhibited greater improvement than the control group. These findings, presented for the first time in the literature through an RCT study, imply the potential benefits of EMDR applications in fibromyalgia treatment.

Fibromyalgia, a condition impacting the quality of life, often requires behavioral and psychotherapeutic approaches in addition to pharmacological treatments (8). This study demonstrated the effectiveness of EMDR therapy in alleviating the pain experienced by individuals with fibromyalgia. Our findings align with case reports indicating the efficacy of EMDR in fibromyalgia cases, a topic explored in limited literature (26, 27). Notably, this study contributes significantly to the literature by presenting results from a randomized controlled trial involving groups with similar sociodemographic characteristics, emphasizing the lasting impact observed in follow-up measurements. Various mechanisms may explain the effectiveness of EMDR therapy in fibromyalgia treatment. Neuroendocrine disorders, particularly those related to the hypothalamic–pituitary–adrenal (HPA) axis, are affected by traumatic experiences and play a role in fibromyalgia development (28). By addressing trauma, EMDR therapy might contribute to the resolution of neuroendocrine disorders, thereby reducing fibromyalgia symptoms. According to the Adaptive Information Processing (AIP) model, traumatic memories stored in multiple dimensions, including physical sensations, contribute to chronic pain. Processing these memories with EMDR can lead to changes in cognitive and emotional dimensions, potentially altering pain pathways (29). Another perspective suggests that fibromyalgia pain could be conceptualized as a repetitive “trauma,” with recurrent emotions and persistent physical symptoms. Reprocessing of these memories with EMDR therapy may contribute to the regression of fibromyalgia symptoms (30). Additionally, desensitization to negative emotions in EMDR therapy may result in a lower pain response, preventing increased limbic activity (27). EMDR therapy can influence the perception of pain by reshaping cognitive, emotional, and physical sensations, making it feel less significant. Considering the rapid effectiveness of EMDR therapy in patients with fibromyalgia, there may be unique and unexplained mechanisms of action at play. It is advised to treat fibromyalgia by addressing depression, as it is important for the clinical prognosis of patients with fibromyalgia (31). There is good evidence that EMDR Therapy can be effective in the treatment of depression (32). In the present study, depressive symptoms significantly decreased after working on traumatic experiences with the EMDR Fibromyalgia Protocol, and this decline remained permanent in the follow-up sessions. The EMDR Fibromyalgia Protocol used in the study was not primarily designed for depression; however, even in this case, the level of depression decreased in the experimental group. We do not know whether the disappearance of depression is dependent on EMDR, the alleviation of pain, or both.

Patients diagnosed with fibromyalgia commonly experience sleep difficulties, including issues with sleep quality, short duration, and challenges in falling asleep and waking up (33). The relationship between sleep quality and fibromyalgia symptoms has been established (34). In this study, both the control and experimental groups exhibited high scores on the Pittsburgh Sleep Quality Index, consistent with existing literature (35, 36). The experimental group showed a significant reduction in sleep difficulties, and this improvement persisted in one-month and three-month follow-ups. It remains unclear whether this enhancement in sleep quality is directly related to the impact of EMDR therapy or to the reduction of pain and depressive symptoms.

Moreover, traumatic stress symptoms in fibromyalgia patients in the experimental group significantly decreased after undergoing the EMDR Fibromyalgia Protocol. This result, coupled with the observed reduction in pain complaints, supports the hypothesis that trauma is a potential etiological factor for fibromyalgia. Addressing traumatic memories not only alleviates fibromyalgia pain, but also contributes to a reduction in depression, sleep problems, and post-traumatic stress levels. Therefore, this study’s hypothesis was confirmed. This aligns with Shapiro’s (11) perspective, which extends the concept of trauma to include “victims of physical illness,” proposing a protocol for chronic diseases and somatic disorders based on the standard EMDR protocol.

This study has certain limitations, including a higher representation of women compared to men, which is, consistent with the existing literature, given that fibromyalgia is more prevalent in women (37). Another limitation is the reliance on self-report scales. In addition, the size of the control group was smaller than that of the experimental group. Unfortunately, various disruptive events in Turkey pose challenges for the research team to reach and retain participants. Moreover, there were a relatively higher number of patients with only primary school education.

## 5 Conclusion

In conclusion, this study indicated that addressing traumatic experiences through EMDR Therapy has the potential to reduce fibromyalgia pain, depression, post-traumatic stress, and sleep difficulties, ultimately enhancing the overall quality of life of individuals with fibromyalgia. Importantly, these positive outcomes persisted after the treatment. Consequently, clinicians are encouraged to consider EMDR therapy as a valuable option for the comprehensive treatment of fibromyalgia patients. Given that fibromyalgia patients often seek help from various healthcare professionals, collaboration with psychiatrists, rheumatologists, and EMDR therapists is recommended. Future research could benefit from replicating this study with a larger participant pool to further validate these findings.

## Data availability statement

The raw data supporting the conclusions of this article will be made available by the authors, without undue reservation.

## Ethics statement

The studies involving humans were approved by Bozyaka Training and Research Hospital Clinical Research Ethics Committee. The studies were conducted in accordance with the local legislation and institutional requirements. The participants provided their written informed consent to participate in this study.

## Author contributions

ZZ: Conceptualization, Data curation, Formal analysis, Funding acquisition, Investigation, Writing – original draft, Writing – review & editing, Methodology, Project administration, Resources, Software, Supervision, Validation, Visualization. DD: Investigation, Methodology, Project administration, Resources, Software, Writing – original draft, Writing – review & editing. TK: Resources, Software, Supervision, Validation, Visualization, Writing – original draft, Writing – review & editing. DG: Investigation, Methodology, Project administration, Resources, Visualization, Writing – original draft. AS: Conceptualization, Data curation, Formal analysis, Resources, Software, Supervision, Validation, Writing – review & editing. HN: Data curation, Funding acquisition, Investigation, Project administration, Validation, Writing – original draft, Writing – review & editing. İC: Conceptualization, Data curation, Investigation, Methodology, Software, Supervision, Writing – review & editing. YA: Formal analysis, Funding acquisition, Project administration, Resources, Validation, Visualization, Writing – original draft. ÖK: Conceptualization, Formal analysis, Investigation, Project administration, Software, Validation, Writing – original draft, Writing – review & editing. CS: Data curation, Funding acquisition, Methodology, Resources, Supervision, Visualization, Writing – original draft, Writing – review & editing. EK: Conceptualization, Data curation, Formal analysis, Funding acquisition, Investigation, Methodology, Project administration, Resources, Software, Supervision, Validation, Visualization, Writing – original draft, Writing – review & editing.
