# Supplementary material for: A randomized controlled trial of Eye Movement Desensitization and Reprocessing (EMDR) Therapy in the treatment of fibromyalgia
Source: Front Psychiatry. 2024 May 21;15:1286118. doi: 10.3389/fpsyt.2024.1286118 (PMC11148358; doi:10.3389/fpsyt.2024.1286118)
Supplement: Supplementary file 1 [file DataSheet_1.pdf]

Fibromyalgia Treatment with EMDR Protocol

1. History of Fibromyalgia

1. *"When did you first begin to have the pain* in your body?"*
2. *"How have your pain experiences developed over time? Is there any change in the intensity? Did they get worse? Did they get better?"*
3. *"How often do you experience pain?"*
4. *"How does your pain affect your social life?"*
5. *"How does your pain affect your family life?"*
6. *"How does your pain affect your work life?"*
7. *"What other problems do you have besides your pain?"*
8. *"Are there any ways that your pain gives you an opportunity NOT to do something that you do not want to do?"*

2. Trauma History

1. "*Were there any traumatic/stressful events that took place relatively close to the **first/earliest time** you had fibromyalgia pain in your body?"* (There could be more than one traumatic memories that coincide with the period before the client's pain onset. Write down all of these and take SUD levels and NCs.)

"*What is the SUD level for __________(*state the memory)."

"What is the negative cognition for ________(state the memory)."

2. "What are the worst traumatic/stressful events related with fibromyalgia pain that took place since the beginning of your pain?"

"*What is the SUD level for __________(*state the memory)."

"*What is the negative cognition for ________(*state the memory)."

3. *"Are there any traumatic/stressful events you recently experienced that triggers your pain?"*

*"What is the SUD level for __________(*state the memory)."

*"What is the negative cognition for ________(*state the memory)."

4. "Are there any other significant traumatic/stressful events related the pain?"

*"What is the SUD level for __________(*state the memory)."

*"What is the negative cognition for ________(*state the memory)."

5. "Are there any significant traumatic/stressful events not related to the pain?"

"What is the SUD level for __________(state the memory)."

"What is the negative cognition for ________(state the memory)."

6. "Are there any traumatic/stressful events not related to the pain before the onset of fibromyalgia pain?"

*"What is the SUD level for __________(*state the memory)."

*"What is the negative cognition for ________(*state the memory)."

* Scan the negative cognitions and check if they had memory network or not. For instance, a negative cognition may come up in different situations and times.

3. Pain as Trauma

1. *"Can you tell me the times when your pain is a painful traumatic memory?"*

Make a list and take SUD and NCs.

*"What is the SUD level for __________(*state the memory)."

*"What is the negative cognition for ________(*state the memory)."

4. Triggers

1. *"Please tell me about your experiences, stressful situations or difficulties that* ***generally trigger*** *your pain/increase the severity of presenting pain or disturb you."*

If the client does not mention the triggers below, you may check for them specifically:

2. "*Have there been any hormonal factors (*e.g. menstrual cycles*) that trigger your pain?"*
3. "*Do certain seasons or changes in the weather affect your pain level*?"
4. "*Do changes in heat, cold and/or humidity affect your pain*?"
5. "*Does over-exertion, illness, injury or traveling affect your pain*?"
6. "*Does noise, or traffic, etc. trigger your pain?"*
7. "*Do certain people in your life affect your pain*? *How?"*
8. "*Do any other events, situations or stimulations trigger your pain*?

5. Treatment Goals

1. *"Please tell me what you would like to achieve and change in your life at the end of this therapy?"*

Or

2*. "Please tell me how would life be for you, if you do not have pain?"*

Note: If the client does not mention pain, dysfunctional relationships around pain, and restricted resources, **ask them.**

Summary Sheet

EMDR Therapist may use the Summary Sheet to ensure that required information is taken.

Diagnosis: ____

Appropriate for EMDR Therapy: Yes No

Past Trauma Memories

Traumatic Events Connected to the Pain NCs SUDs

Earliest : ____

Worst : ____

Most Recent: ____

Other Significant Traumatic Events Related to FMS: ____________________

Other Significant Traumatic Events Not Related to FMS: ____________________

Other Traumatic/Stressful Events in Life before Pain

NCs SUDs

____

____

____

____

____

____

Traumatic/Stressful Pain Experiences: Pain as Trauma NCs SUDs

Earliest : ____

Worst : ____

Most Recent: ____

Other Significant Pain Experiences: ______________________________________

Present Triggers

Triggers Initiating or Increasing the Pain

(most frequent to less frequent) SUDs

____

____

Treatment Goals

____

____

Future Template

After the therapy ends, a Future Template based on triggers.

Treatment Plan

Take Summary Sheet as a guide and use this hierarchy to structure EMDR past trauma processing:

1. First Memory Related to First Fibromyalgia Pain

2. Other Memories Related to Fibromyalgia Pain

3. Fibromyalgia Pain as Trauma

4. Triggers: Working With Triggers

5. Unrelated Memories Before The Fibromyalgia Onset: After making a general assessment about the treatment process, if therapist and client agree working on the memories before the fibromyalgia onset, they may focus on them.

When you begin to work with EMDR, use Standard EMDR Protocol. Follow the sequence in the "Treatment Plan" to target the memories. During the EMDR sessions if any new traumatic experience comes up, check the strength of its connection with patient's fibromyalgia pain and then decide where to put the new experience in the hierarchy. This means that during the course of the treatment the case planning is subject to change at any time.

** Fibromyalgia Pain*

Use the following assessment for each memory.

Incident

"Focus on the__________(state the issue\event)"

Picture

"What picture represents the most traumatic part of the entire incident________ (state the issue)*?"*

Negative Cognition (NC)

"What words best go with the picture that express your negative belief about yourself now?"

Positive Cognition (PC)

"When you bring up that picture or__________(state the issue), *what would you like to believe about yourself now?"*

Validity of Cognition (VoC)

"When you think of__________(state the issue, or picture), *how true do those words__________*(clinician repeats the positive cognition) *feel to you now on a scale of 1 to 7, where 1 feels completely false and 7 feels completely true?"*

1 2 3 4 5 6 7

(completely false) (completely true)

Sometimes, it is necessary to explain further.

*"Remember, sometimes we know something with our head, but it feels differently in our gut. In this case, what is the gut-level feeling of the truth of*__________(clinician state the positive cognition), *from 1* (completely false) *to 7* (completely true) *?"*

1 2 3 4 5 6 7

(completely false) (completely true)

Emotions

*"When you bring up the picture*__________(state the issue) *and those words* __________ (clinician states the negative cognition), *what emotion do you feel now?"*

Subjective Units of Disturbance (SUD)

"On a scale of 0 to 10, where 0 is no disturbance or neutral and 10 is the highest disturbance you can imagine, how disturbing does it feel now?"

0 1 2 3 4 5 6 7 8 9 10

(no disturbance) (highest disturbance)

Location of Body Sensation

"Where do you feel it (the disturbance) *in your body?"*

Use Phases 4 through 8 for each incident. Work with the past issues, checking to see if the other targets have been processed or still need to be processed with the Standard EMDR Protocol. Continue to process the past issues until they are no longer an issue.
